# A new record of the spider family Caponiidae from China (Arachnida, Araneae)

**DOI:** 10.3897/zookeys.851.33351

**Published:** 2019-06-03

**Authors:** Ke-ke Liu, Hai-qiang Yin, Ji-he Liu, Xiang Xu, Yong-hong Xiao, Xian-jin Peng

**Affiliations:** 1 College of Life Science, Hunan Normal University, Changsha 410081, Hunan, China; 2 College of Life Science, Jinggangshan University, Ji’an 343009, Jiangxi, China; 3 The National & Local Joint Engineering Laboratory of Animal Peptide Drug Development (Hunan Normal University), National Development and Reform Commission, Changsha, Hunan 410081, China

**Keywords:** Distribution, Guangxi Zhuang Autonomous Region, *
Laoponia
*, taxonomy

## Abstract

The family Caponiidae Simon, 1890 is reported for the first time from China. The total number of the known spider families from China increases to 72 with the addition of this family newly recorded in the present paper. Based on male and female specimens collected from Guangxi, China, *Laoponiasaetosa* Platnick & Jäger, 2008 is illustrated and a global distribution map is generated.

## Introduction

The family Caponiidae is a group of Haplogynae ecribellate spiders with a patchy but nearly global distribution, typically found on the ground, under rocks, and in burrows and leaf litter. At present, the known caponiids are mainly distributed in America and Africa, absent from Australia and New Zealand, with only three species found in Asia (World Spider Catalog 2019). The first Asian spiders of the family Caponiidae were found in Laos in 2007 by Peter Jäger who collected them using the sieving method; based on these specimens, Platnick and Jäger (2008) established the genus *Laoponia* to contain the first member of the family Caponiidae discovered in Asia: *Laoponiasaetosa*, which was also found subsequently in Vietnam (Liu et al. 2010). *Laoponiasaetosa* is characterized by having two eyes, a short embolus, an absence of a tegular apophysis on the male palp, normal endites, and a single receptaculum in the female genitalia (Platnick and Jäger 2008; Liu et al. 2010). The second and third Caponiidae species known to occur in Asia are *Iraponiascutata* Kranz-Baltensperger, Platnick & Dupérré, 2009 from Iran and *Laoponiapseudosaetosa* Liu, Li & Pham, 2010 from Vietnam.

Caponiidae consists of 119 species classified into 18 genera worldwide (World Spider Catalog 2019). Recently, more details based on the morphological characters of the genus *Medionops* and *Nops* were revealed by Sánchez-Ruiz (2017) and Brescovit (2018). Many structures of the legs and genitalia were defined as the diagnostic structures which are undoubtedly beneficial to future taxonomic studies (Sánchez-Ruiz and Brescovit 2017, 2018). With the discovery of many unusual characters, such as a male palp with anterior curvature and female genitalia with lateral extensions of the posterior plate, the caponiids are undergoing re-evaluation and re-organization (Dupérré 2014; Sánchez-Ruiz et al. 2015; Sánchez-Ruiz and Brescovit 2017).

During the past four years, several explorations to Guangxi Zhuang Autonomous Region, China, have been made by the authors, and many spider specimens were collected. Among the many specimens from Guangxi, only four specimens were identified as *Laoponiasaetosa*. It is the first member of the family Caponiidae to be found in China.

It isn’t surprising to find *Laoponiasaetosa* in some areas of Guangxi, China which is very near Vietnam. After all this species has been reported in Vietnam. What is interesting is that in southwest Guangxi, three National Nature Reserves located in Chongzuo City (Nonggang National Nature Reserve, Chongzuo White-headed langur National Nature Reserve) and Nanning City (Damingshan National Nature Reserve) have been explored by authors, but it is only in Nonggang National Nature Reserve that *L.saetosa* was collected. There are only two possible reasons for this. One possibility is that *Laoponia* species have very narrow distribution regions in China and they distribute only in the places very near Vietnam. Geographically, Nonggang National Nature Reserve (22°13'56"−22°39'09"N, 106°42'28"−107°04'54"E) is actually closer to Vietnam than both Chongzuo White-headed langur National Nature Reserve (22°10'43"−22°36'55"N, 107°16'53"−107°59'46"E) and Damingshan National Nature Reserve (23°24'−23°30'N, 108°20'−108°24'E). The other possibility is that besides Nonggang, the other regions of Guangxi are also potential distribution regions of *Laoponia* and collectors simply haven’t found them yet. However, the reasons why collectors didn’t collect them are very complex and are related with at least two factors: time and methods.

The *Laoponia* specimens examined by Liu et al. (2010) were collected during an entire year-long sampling period (1 April 2014 – 31 March 2015) and it is hard to say which season is the peak abundance for this species. The *Laoponia* specimens examined by Platnick and Jäger (2008) and Jäger and Praxaysombath (2009) were collected in February/March (the dry season in Laos). The specimens in the present study were collected in between October and November (the dry season in southern China). It is possible that from October to March or April of the next year is the mating period of *L.saetosa* and adult specimens are relatively easily found. It seems that this species is inclined to live the dry circumstance and it is also very possible it has a capability for drought resistance.

Caponiids are wandering hunters. According to the previous description of *L.saetosa* and the authors’ collecting experience, it is usually by sieving leaf litter or using pitfall traps that this species was successfully collected.

The authors made an exploration in Damingshan National Nature Reserve, Nanning City, Guangxi Zhuang Autonomous Region, China in November (dry season), 2018 and sieving leaf litter was widely used. The authors also made an exploration in Chongzuo White-headed langur National Nature Reserve, Chongzuo City, Guangxi Zhuang Autonomous Region, China in between August and September (rainy season), 2015 and sieving leaf litter was rarely used during exploration because of rainy weather. Though no specimens of *L.saetosa* were collected in both explorations, the authors think, based on the combinations of geographic location, time and methods, it is very possible that Damingshan National Nature Reserve isn’t the distribute region of *L.saetosa*, while it is also highly probable that *L.saetosa* can be collected in Chongzuo White-headed langur National Nature Reserve in suitable season by using suitable collecting methods. Certainly, this must to be confirmed by future collecting and further research from Guangxi, China.

Though *L.saetosa* has been described by Platnick and Jäger (2008) and redescribed by Liu et al. (2010), there are also shortcomings in previous taxonomic treatments of this species. The embolus was broken and its distal end was missing in SEM photographs supplied by Platnick and Jäger in the original description. Liu et al. took only a photograph for one side of male palp (only retrolateral view) and omitted at least one standard anatomical view. The present paper supplies more digital color and SEM images based on the intact specimens. The presentation of more detailed features in images makes it more easily to distinguish *Laoponia* from the other genera such as *Iraponia*, *Nops*, *Nopsides* and *Tarsonops* in family Caponiidae.

Here this newly recorded spider is reported from China and illustrated in detail, and a global distribution map of *Laoponiasaetosa* is also provided. The total number of spider families known from China increases to 72 with the addition of this record.

## Materials and methods

Specimens were examined and photographed using a LEICA M205C stereomicroscope with a LEICA MC170 HD. Both the male palps and female genitalia were detached from the spider’s body and observed in 75% ethanol. For the photographs of the female vulva, the specimens were previously digested with pancreatin and cleaned after 2 h of digestion. Specimens including detached copulatory organs were stored in 75% ethanol after examination. All the specimens are deposited in Hunan Normal University (HNU), Changsha.

All morphological measurements were calculated using a stereomicroscope (LEICA M205C) and are given in millimeters. Leg measurements are given as total length (femur, patella, tibia, metatarsus, tarsus).

That leg I and IV, not leg II and III were supplied with SEM photographs is following the references on caponiids (Sánchez-Ruiz 2017; Brescovit 2018; Sánchez-Ruiz and Brescovit 2017; 2018). Leg I and IV have more important characteristics than Leg II and III.

Terminology of the male and female genitalia follows Sánchez-Ruiz et al. (2015) and Sánchez-Ruiz and Brescovit (2017). The abbreviations used in the present paper are as follows:

**AL** abdomen length;

**Ar** arolium;

**AP** anterior plate;

**AW** abdomen width;

**Bu** bulb;

**CL** carapace length;

**CW** carapace width;

**CWLNNR** Chongzuo White-headed langur National Nature Reserve;

**DMR** distal margin of receptaculum;

**DNNR** Damingshan National Nature Reserve;

**Em** embolus;

**ESS** external sclerotization around spiracles;

**Fu** furrow;

**LEP** lateral extensions of posterior plate;

**LO** lyriform organs;

**MC** median concavity;

**MS** membranous sac;

**MTS** metatarsal stopper furrow;

**NNNR** Nonggang National Nature Reserve;

**PP** posterior plate;

**Re** receptaculum;

**SDO** sperm duct opening;

**SS** slit sensillum;

**TT** tracheal trunk.

## Taxonomy

### Family Caponiidae Simon, 1890

#### Genus Laoponia

Taxon classificationAnimaliaAraneaeCaponiidae

Platnick & Jäger, 2008

Laoponia : Platnick and Jäger 2008: 2, figs 1−25.

##### Type species.

*Laoponiasaetosa* Platnick & Jäger, 2008: 4, figs 1−25, 31−34, type locality Laos.

##### Diagnosis.

*Laoponia* species is similar to *Nops* species (see Sánchez-Ruiz and Brescovit 2015: 133, fig. 6; 135, fig. 18) and *Tarsonops* species (see Sánchez-Ruiz et al. 2015: 47, fig. 15; Sánchez-Ruiz and Brescovit 2018, 62, fig. 38H) in having a slit sensillum at the base of fan on chelicerae and the lyriform organ distally located at metatarsi, but can be distinguished from other genera by the relatively shorter, slender embolus with a sharp tip in males (wide, membranous in *Nopsides* Chamberlin, 1924; broad, ribbon-shaped in *Iraponia* Kranz-Baltensperger, Platnick & Dupérré, 2009; with small extensions on tip or a sclerotized margin in *Nops* MacLeay, 1839), and the normal legs without some appendages (such as the median translucent ventral longitudinal keel, the translucent extension of the membrane, subsegmented tarsi, and the presence of a gladius between the anterior metatarsi and tarsi in *Nops* MacLeay, 1839) (Platnick and Jäger 2008; Liu et al. 2010; Jiménez et al. 2011; Sánchez-Ruiz and Brescovit 2018). Female internal genitalia with clearly sclerotized distal margin and simple invagination of receptaculum and relatively wide base of uterus externus (narrow in *Iraponia* Kranz-Baltensperger, Platnick and Dupérré 2009). Unfortunately, there are only two *Laoponia* species reported worldwide: one is known with both male and female specimens and the other only with male specimens. More information on the females of this genus is needed.

#### Laoponia saetosa

Taxon classificationAnimaliaAraneaeCaponiidae

Platnick & Jäger, 2008

Figs 1 , 2 , 3 , 4 , 5 , 6 , 7 , 8

Laoponia saetosa Platnick & Jäger, 2008: 4, figs 1−25, 31−34; Jäger and Praxaysombath, 2009: 31, figs 7−14; Liu, Li and Pham, 2010: 22, figs 1−5, 9, 11−12, 14.

##### Material examined.

All specimens examined in this study were collected by Ailan He, Keke Liu, Qu Cai, Jihe Liu, Jinxin Liu, and Zongguang Huang from Nonggang National Nature Reserve, Guangxi, China. 1 ♀, Longzhou County, Nonggang Station, the entrance of core area, 22°27'50.94"N, 106°55'56.58"E, 230 m, leaf litter, 26.X.2017; 1 ♂, Longzhou County, Sanlian Station, 22°32'4.93"N, 106°50'13.07"E, 310 m, leaf litter, 30.X.2017; 1 ♀, Longzhou County, Xiangshui Station, 18^th^ boundary tablet, 22°26'8.38"N, 107°1'26.37"E, 260 m, leaf litter, 1.XI.2017; 1 ♂, Ningming County, Longrui Station, Huashan National Village, the road behind the hill, 22°14'29.45"N, 107°3'32.01"E, 260 m, leaf litter, 4.XI.2017.

**Figure 1.**
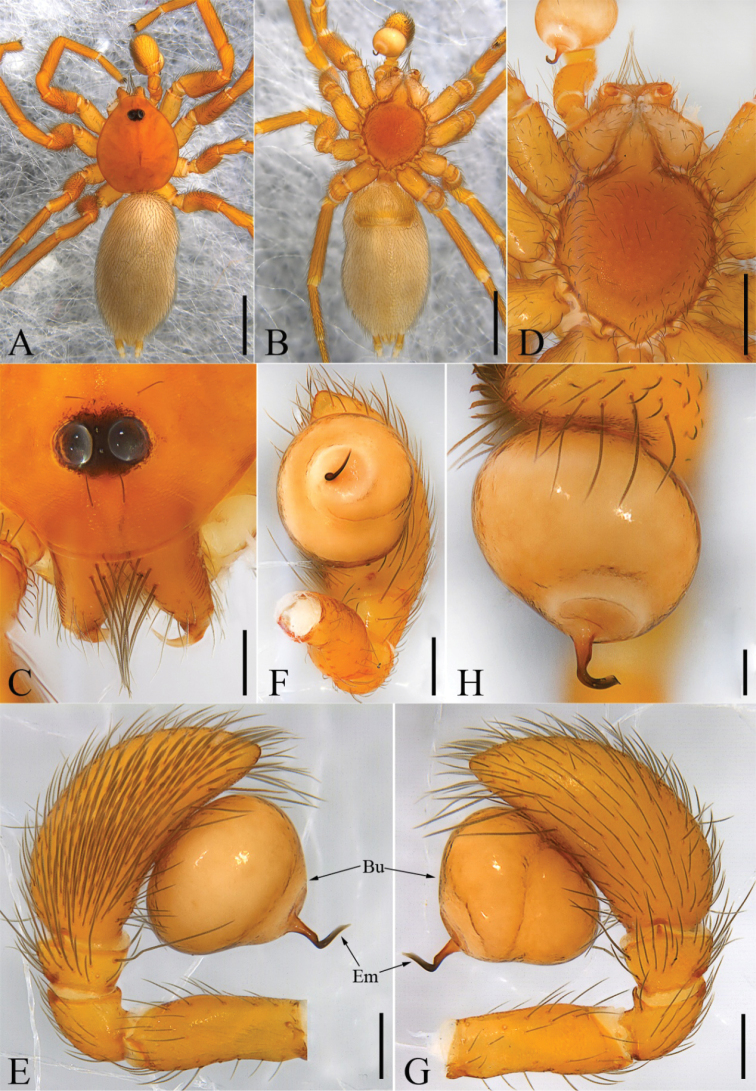
*Laoponiasaetosa* Platnick & Jäger, 2008, male **A** habitus, dorsal view **B** habitus, ventral view **C** prosoma, frontal view, slightly lateral **D** prosoma, ventral view **E** palp, prolateral view **F** palp, left, ventral view **G** palp, retrolateral view **H** palp, dorsal view. Scale bars: 1 mm (**A, B**); 0.2 mm (**C, E–G**); 0.5 mm (**D**); 0.1 mm (**H**).

##### Diagnosis.

The male of this species resembles that of *L.pseudosaetosa* Liu, Li & Pham, 2010 in having short, slender embolus with a sharp tip and entire legs without translucent ventral keel on metatarsi (see Liu et al. 2010: 24, figs 10, 13), but can be distinguished by a stout bulb and the embolus with a distinctively strongly curved median part (Figs 1E−H, 5). The female can be easily distinguished from another Asian species, *Iraponiascutata* Kranz-Baltensperger, Platnick & Dupérré, 2009, by the presence of only two eyes (six eyes in *I.scutata*), abdomen lacking the postepigastric scutum, the shape of median membranous sac (large in *I.scutata*), and relatively wide base of the uterus externus (narrow in *I.scutata*) (Kranz-Baltensperger et al. 2009).

**Figure 2.**
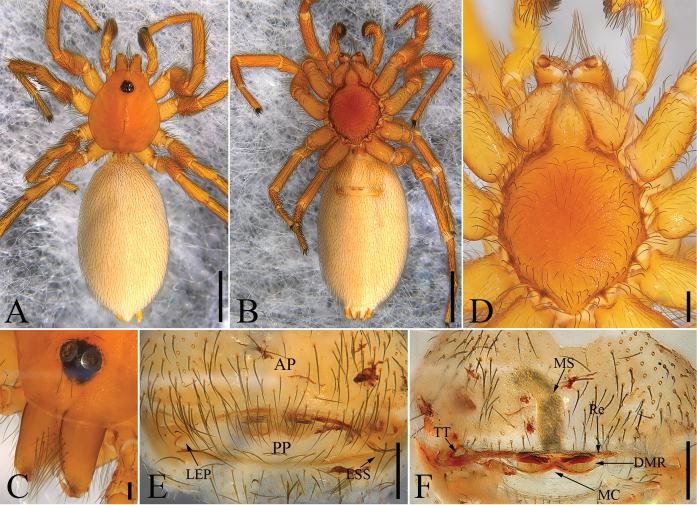
*Laoponiasaetosa* Platnick & Jäger, 2008, female **A** habitus, dorsal view **B** habitus, ventral view **C** prosoma, frontal view, slightly lateral **D** prosoma, ventral view **E** epigyne, ventral view **F** vulva, dorsal view. Scale bars: 1 mm (**A, B**); 0.1 mm (**C**); 0.2 mm (**D–F**).

##### Description.

**Figure 3.**
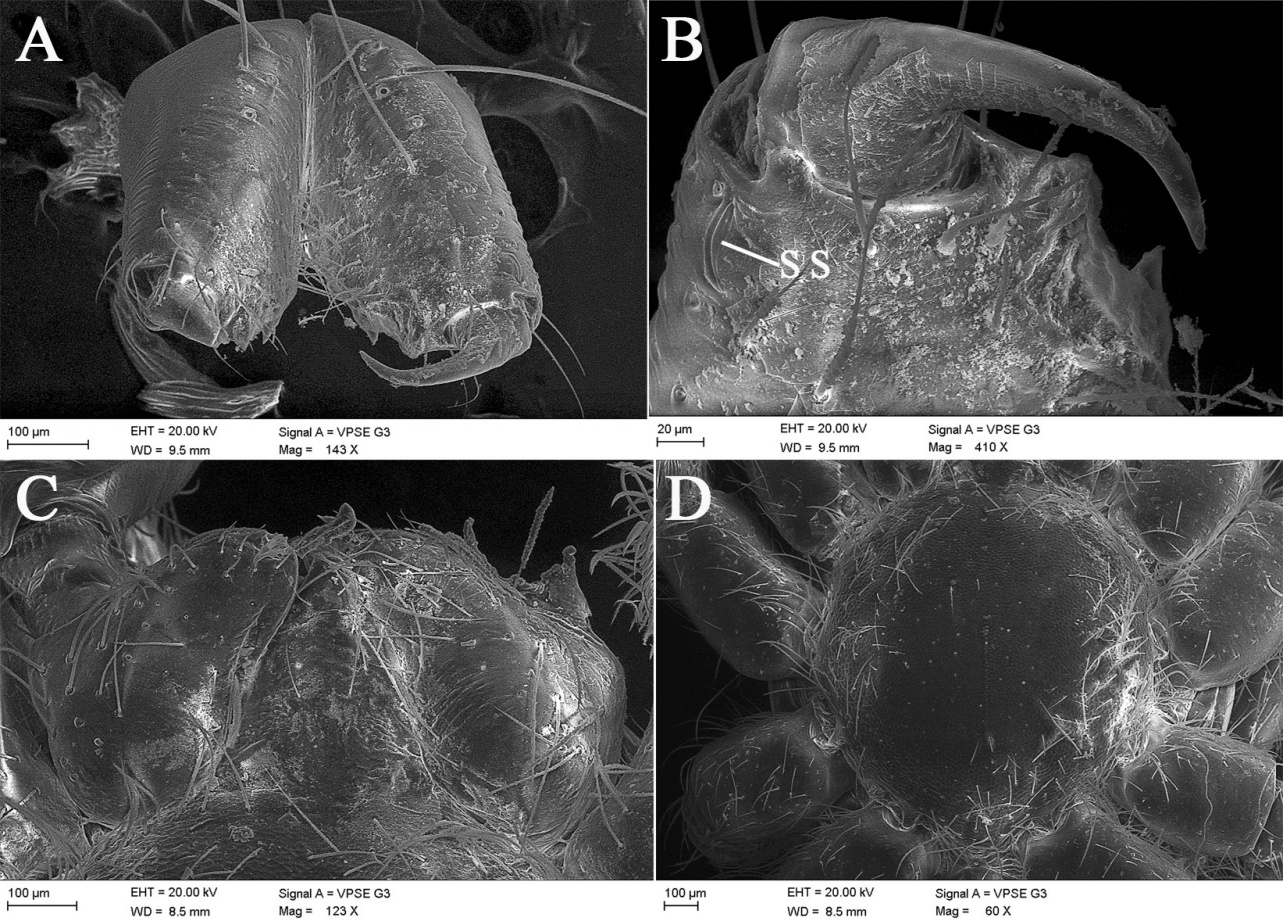
*Laoponiasaetosa* Platnick & Jäger, 2008, male mouthpart and sternum **A** chelicerae, frontal view **B** left chelicera, frontal view **C** endites and labium, ventral view **D** sternum, ventral view.

**Figure 4.**
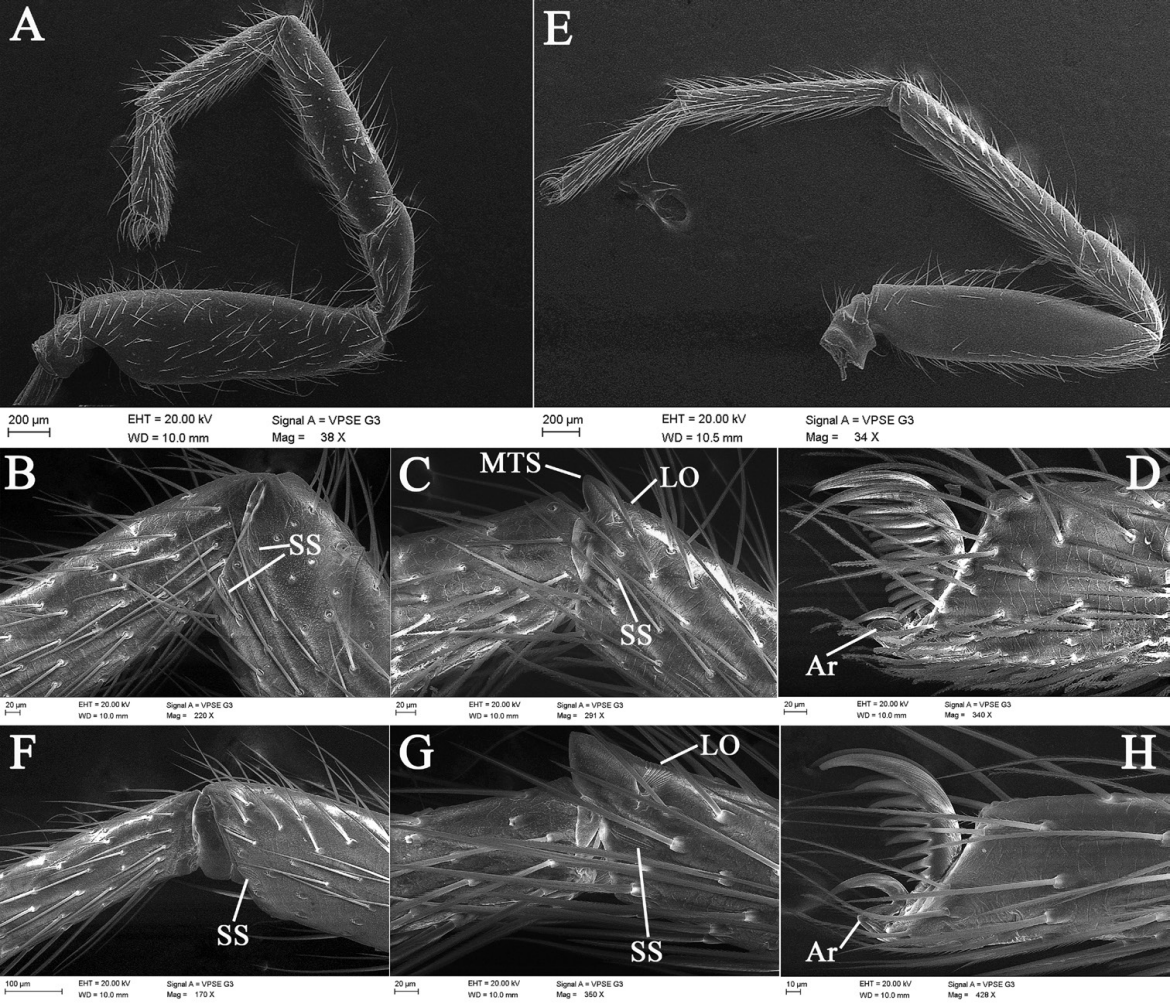
*Laoponiasaetosa* Platnick & Jäger, 2008, male legs **A** left leg I, retrolateral view **B** same, join of tibia and metatarsus, retrolateral view **C** same, join of tarsus and metatarsus, retrolateral view **D** same, tarsal claws, retrolateral view **E** left leg IV, retrolateral view **F** same, join of tibia and metatarsus, retrolateral view **G** same, join of tarsus and metatarsus, retrolateral view **H** same, tarsal claws, retrolateral view.

**Figure 5.**
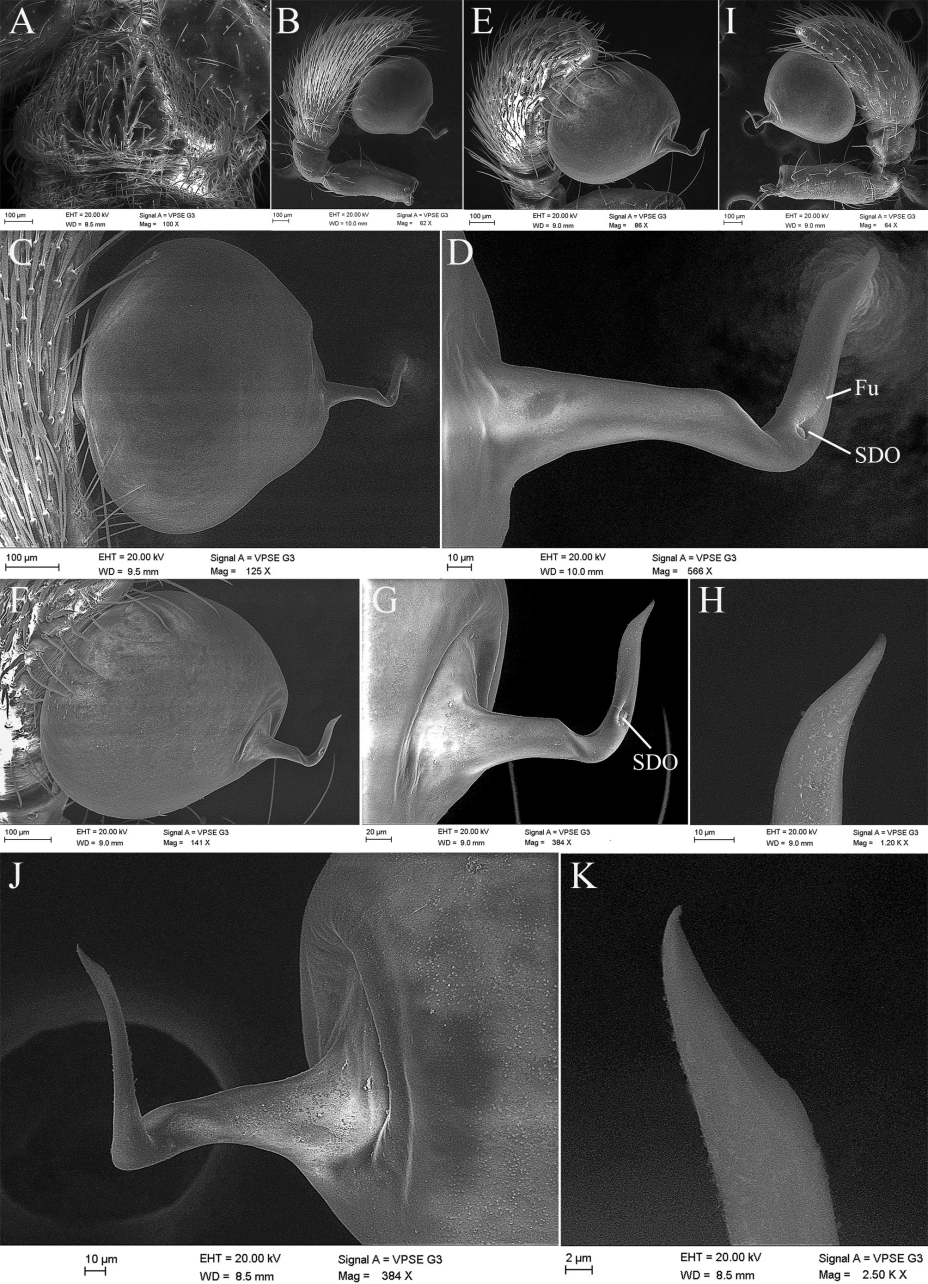
*Laoponiasaetosa* Platnick & Jäger, 2008, male genital area and left palp **A** genital area, ventral view **B** palp, prolateral view **C** same, detail of bulb and embolus **D** same, detail of embolus **E** dorsal view, slightly prolateral **F** same, detail of bulb and embolus **G** same, detail of embolus **H** same, the apex of embolus **I** retrolateral view **J** same, detail of bulb and embolus **K** same, the apex of embolus.

Male. Habitus as in Fig. 1A, B. Total length 4.37. Carapace (Fig. 1A) length 1.66, width 1.39, orange, broadly oval in dorsal view (CW/CL ≈ 0.84), pars cephalica slightly domed in lateral view, anteriorly narrowed to 0.3 times its maximum width, with abundant scale-shaped lattices on the surface, indistinct cervical groove and distinct fovea. Eyes (Fig. 1C): circular, with dark pigment around them; diameters 0.14; interdistance 0.05, separated by about one third their diameters; with a group of setae in front of and behind eyes respectively. Mouthparts (Figs 1B, 3A−C): chelicerae straight, light orange, each with an apical group of 6−10 strong and converging setae from basal to median part in frontal view; median lamina long, with dark, heavily sclerotized tip in the furrow; a membranous lobe located at the median part between lamina and fang base; the base of fan with a slit sensillum laterally in frontal view; stridulatory ridges clear, covering more than 1/2 of prolateral surface; endites convergent anteriorly but not touching, light orange, anteriorly with a relatively long membranous projection, serrula present, tiny, middle part wider than distal and proximal margins and forming an obtuse angle of about 100 degrees, covered with scattered long setae from median to posterior surface; labium almost diamond-shaped, fused to sternum, anterior surface of labium with long, longitudinal, submarginally sclerotized strips and the apical part narrow, a bit round. Sternum (Fig. 1D, 3D) slightly longer than wide, ovoid, with abundant setae and fine reticular lines around the edge, orange, darker at the edge. Pedicel, short, smooth, without setae on the surface. Abdomen (Figs 1A, B, 5A) uniformly white, length 2.62, width 1.48, strongly elongate oval in dorsal view (AW/AL ≈ 0.56), with abundant setae dorsally and two pairs of respiratory spiracles clustered around epigastric groove. Legs (Figs 1A, B, 4) orange, with abundant setae, the base of femur thickened, without sub-segmentation or membranous processes and strong spines on each segment; femora enlarged anteriorly; patella shorter than femora, tibia, metatarsus, and tarsus in legs I, III, IV; the distal part of tibia with few slit sensilla in retrolateral view; metatarsi entire, with obvious metatarsal dorsal stopper and lyriform organ distally in retrolateral view; tarsus with three claws: paired claws with 8−10 teeth, gradually enlarged from base to apex; the unpaired claw small, associated with a relatively small arolium at the base; tibia, metatarsus, and tarsi I−IV with trichobothria in a single row in retrolateral view; leg measurements in mm: I 3.77 (0.92, 0.60, 0.85, 0.79, 0.61); II 3.43 (0.72, 0.65, 0.98, 0.70, 0.38); III 3.30 (0.66, 0.45, 0.89, 0.74, 0.56); IV 4.43 (0.80, 0.69, 1.29, 0.91, 0.74). Leg formula: 4123. Six spinnerets with abundant setae, posterior laterals with two segments.

Genitalia (Figs 1A, B, D−H, 5): epigastric region with faint scutum, furrow broad, slightly bent, slit-shaped with rebordered margins, situated at level of bi-lateral spiracles; palp stout, yellow; patella and tibia short, patella slightly longer than tibia but shorter than femur; cymbium ovoid, with thickened setae at the distal area; bulb light orange, ovoid, 1.2 times as long as its maximum width in frontal view, medially with a constriction in retrolateral view, ventrally with small circular area clearly delimited from remainder of cuticle; embolus sclerotized, hook-shaped, twisted medially, including basal and distal part, apex bent outward in frontal view, forming an angle of approximately 100° with the axis of basal part, with a sperm duct opening associated with a shallow furrow extending towards the apical part.

##### Female.

**Figure 6.**
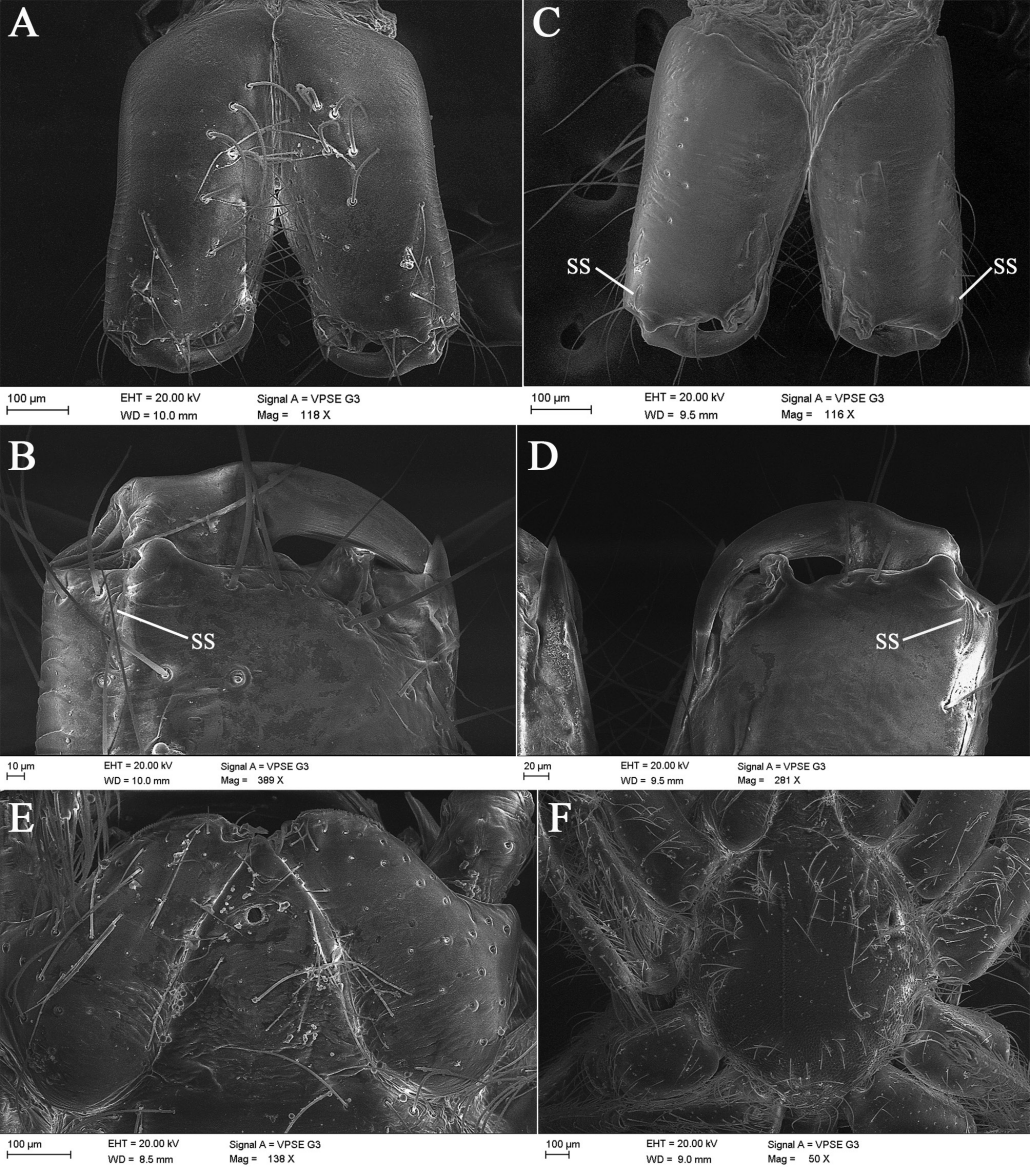
*Laoponiasaetosa* Platnick & Jäger, 2008, female mouthpart and sternum **A** chelicerae, frontal view **B** left chelicera, frontal view **C** chelicerae, posterior view **D** left chelicera, frontal view **E** endites and labium, ventral view **F** sternum, ventral view.

**Figure 7.**
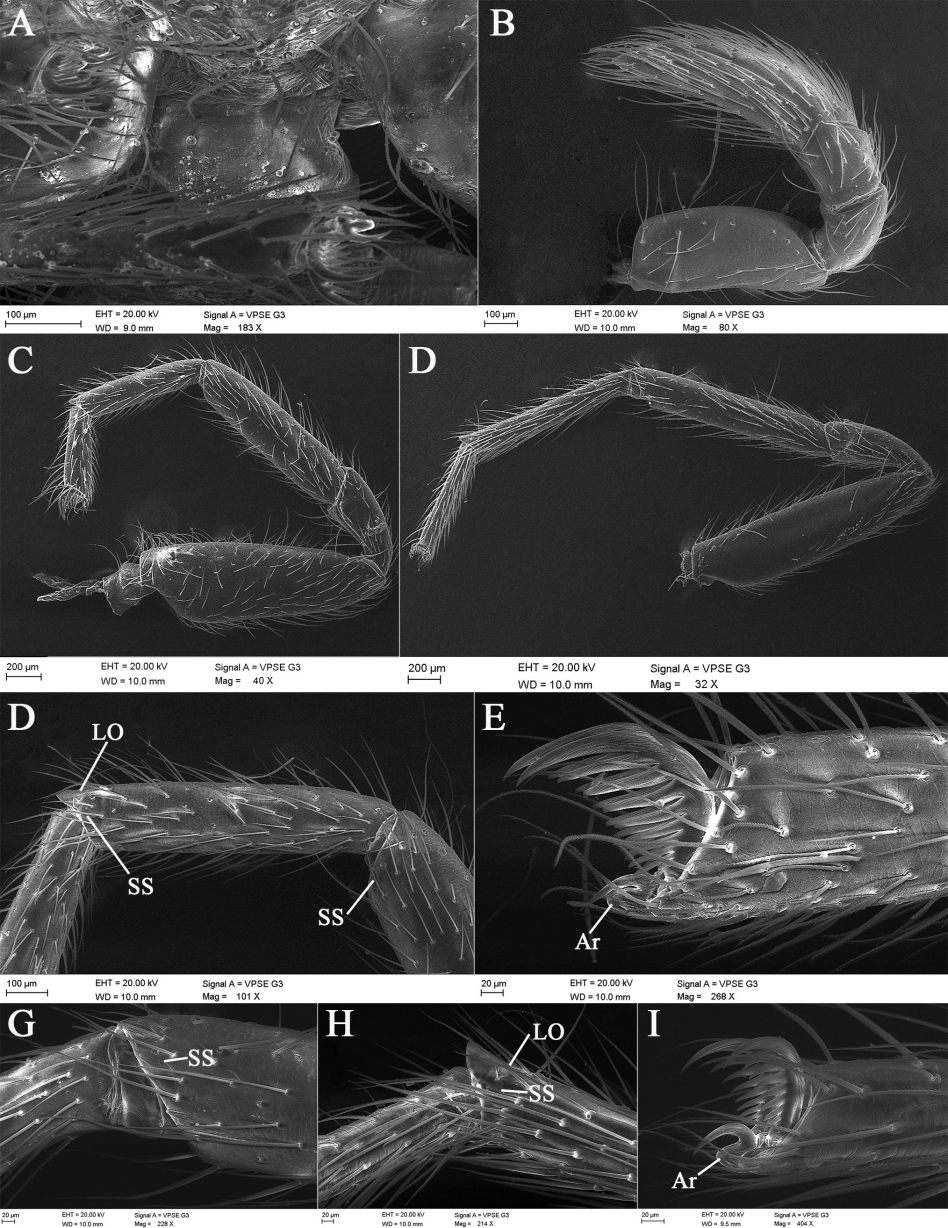
*Laoponiasaetosa* Platnick & Jäger, 2008, female **A** pedicel, ventral view **B** left palp, retrolateral view **C** left leg I, retrolateral view **D** same, join of tibia and metatarsus, and patella and tibia, retrolateral view **E** same, tarsal claws, retrolateral view **F** left leg IV, retrolateral view **G** same, join of tibia and metatarsus, retrolateral view **H** same, join of tarsus and metatarsus, retrolateral view **I** same, tarsal claws, retrolateral view.

Habitus as in Fig. 2A, B. As in male except as follows. Total length 4.98, carapace length 1.67, width 1.43, broadly oval in dorsal view (CW/CL ≈ 0.86), anteriorly narrowed to 0.4 times its maximum width. Eyes (Fig. 2C), diameters: 0.14; interdistances: 0.04. Mouthparts (Figs 2B, D, 6A−E): labium with a round hollow anteriorly. Abdomen (Fig. 2A, B), length 3.19, width 1.79, strongly elongate oval in dorsal view (AW/AL ≈ 0.56). Palp (Fig. 7B) and tarsus longer than patella plus tibia, with dense setae. Leg (Figs 2A, B, 7C−I) measurements in mm: I 3.48 (0.67, 0.57, 1.04, 0.61, 0.59); II 3.37 (0.68, 0.61, 0.65, 0.84, 0.59); III 3.27 (0.59, 0.54, 0.74, 0.70, 0.60); IV 4.62 (0.80, 0.76, 1.33, 0.95, 0.78).

Female genitalia (Fig. 2E, F). In ventral view (Fig. 2E), anterior plate distinct, almost reaching proximal part of abdomen; external sclerotization of spiracles yellowish, small, near the lateral extensions of posterior plate; posterior plate oval. In dorsal view (Fig. 2F), tracheal trunk long, anteriorly directed, extending anteriorly as long as cone-shaped membranous sac, membranous sac located on the receptaculum; with a thin, convex and sclerotized distal margin of receptaculum; postero-medially with distinct median concavity.

##### Distribution.

Laos (Luang Prabang Province), Vietnam (Ninh Binh Province, Hai Phong Province), China (Guangxi Zhuang Autonomous Region).

##### Remarks.

The female genitalia consist of a single receptaculum and a cone-shaped membranous sac in life. The tracheal trunks of the female specimens examined in this study were broken off during preparation. The illustrations of this species were examined by Peter Jäger who first collected and described this species, and who confirmed the identification.

**Figure 8.**
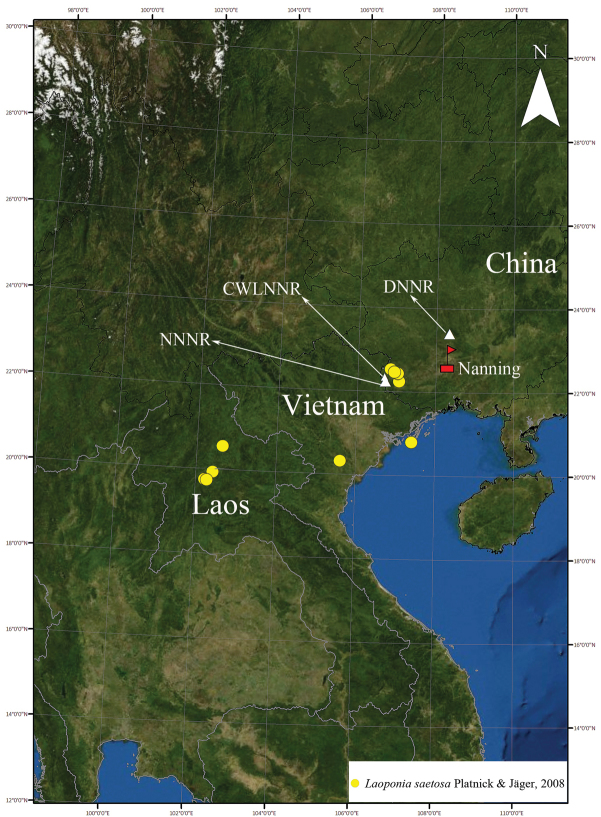
Localities of records of *Laoponiasaetosa* Platnick & Jäger, 2008 in the world (see World Spider Catalog 2019 and Material examined for details).

## Supplementary Material

XML Treatment for Laoponia

XML Treatment for Laoponia saetosa
